# Tau imaging in neurodegenerative diseases

**DOI:** 10.1007/s00259-015-3231-2

**Published:** 2015-11-16

**Authors:** M. Dani, D. J. Brooks, P. Edison

**Affiliations:** Neurology Imaging Unit, Division of Neuroscience, Imperial College London, 1st Floor, B Block, Hammersmith Hospital, Du Cane Road, London, W12 0NN UK; Institute of Clinical Medicine, Aarhus University, Aarhus, Denmark

**Keywords:** Tau imaging, Dementia, Neurodegenerative diseases

## Abstract

Aggregated tau protein is a major neuropathological substrate central to the pathophysiology of neurodegenerative diseases such as Alzheimer’s disease (AD), frontotemporal dementia, progressive supranuclear palsy, corticobasal degeneration and chronic traumatic encephalopathy. In AD, it has been shown that the density of hyperphosphorylated tau tangles correlates closely with neuronal dysfunction and cell death, unlike β-amyloid. Until now, diagnostic and pathologic information about tau deposition has only been available from invasive techniques such as brain biopsy or autopsy. The recent development of selective in-vivo tau PET imaging ligands including [^18^F]THK523, [^18^F]THK5117, [^18^F]THK5105 and [^18^F]THK5351, [^18^F]AV1451(T807) and [^11^C]PBB3 has provided information about the role of tau in the early phases of neurodegenerative diseases, and provided support for diagnosis, prognosis, and imaging biomarkers to track disease progression. Moreover, the spatial and longitudinal relationship of tau distribution compared with β - amyloid and other pathologies in these diseases can be mapped. In this review, we discuss the role of aggregated tau in tauopathies, the challenges posed in developing selective tau ligands as biomarkers, the state of development in tau tracers, and the new clinical information that has been uncovered, as well as the opportunities for improving diagnosis and designing clinical trials in the future.

## Introduction

Alzheimer’s disease (AD), Parkinson’s disease without (PD) and with later dementia (PDD), Lewy body dementia (LBD), frontotemporal dementia (FTD), and corticobasal degeneration (CBD) are common neurodegenerative disorders. Tau is a microtubule-associated protein which is essential for neuronal stability and transport of axonal nutrients. Aggregated tau, due to hyperphosphorylation, is a pathological characteristic of a group of neurodegenerative conditions known as the tauopathies [1]. The neuropathological substrates of AD are tau neurofibrillary tangles (NFT) and β-amyloid (Aβ) plaques, while activated microglia, astrocytes, and neuropil threads also play a significant role in disease pathogenesis. It has been shown that Aβ plaque deposition can begin decades before symptom onset, while tau deposition is more closely associated with symptom onset due to neuronal dysfunction, its levels at autopsy correlating well pre-morbid cognitive status [2, 3].

Recent advances in selective tau tracer development for positron emission tomography (PET) imaging have, for the first time, allowed in-vivo exploration of the presence and extent of tau pathology in patients suspected of having tauopathies. Clinically, tau PET imaging can provide valuable support in the early differential diagnosis of neurodegenerative disorders by revealing whether a characteristic pattern of aggregated tau is present. It also provides a potential biomarker of disease progression. Over the next decade, tau imaging is likely to dominate the field of dementia research and, in this review, we will discuss current developments in novel tau tracers, future applications, and how this could extend our knowledge of dementia.

## Tau protein and its role in the pathophysiology of the tauopathies

Tau is a natively unfolded phosphorylated protein that is present mainly in axons, and binds to microtubules, stabilizing them. Microtubules comprise the cell cytoskeleton, and are critical for maintaining the structural integrity of the cell and for transporting nutrients from the soma down the axons to synaptic terminals [1, 4, 5]. Tau protein exists as six distinct isoforms that result from alternate mRNA splicing of the MAPT (microtubule associated protein tau) gene on chromosome 17 (cytogenetic location 17q21.1). The isoforms differ in the number of microtubule binding repeats (which are encoded by exon 10) that are present, and tau can exist in 3-repeat (3R) or 4-repeat (4R) forms. The healthy adult human cortex has equal numbers of 3R and 4R isoforms, and its tau expression is roughly double that seen in the white matter and cerebellum [1]. Through the tandem repeats, tau assembles into filaments which have a cross β structure, similar to that of Aβ. The ability of tau to bind to microtubules is also regulated by post-translational modification of the protein by phosphorylation, glycosylation, glycation, ubiquitination, sumoylation, and nitration [1, 6]. The functions of tau are regulated in part by its phosphorylation state, and the protein has multiple kinase phosphorylation sites [7, 8].

In all neurodegenerative diseases in which tau is implicated, it is in a hyperphosphorylated form, and this is responsible for its aggregation, leading to neuronal dysfunction and death. Hyperphosphorylation prevents tau binding to microtubules, reducing their stability, which in turn leads to impaired axon transport. Aggregated tau in AD exists as paired helical filaments which further coalesce into neurofibrillary tangles (NFTs) [5, 9, 10]. These then lead to impaired synaptic and neuronal dysfunction [11].

In AD, the distribution of extracellular Aβ plaques can be variable between individuals, but amyloid deposition is thought to start in the inferior frontal cingulate areas and then spread to association cortex [12]. According to Braak staging of AD, the deposition of NFTs follows a more predictable and stereotyped course than Aβ as the disease progresses [12]. NFTs are first detected in the transentorhinal cortex (stages 1 and 2) in the presymptomatic stage of AD, and then spread to the limbic areas, by which time symptoms become evident. NFTs finally involve association cortical areas by stages 5–6, when symptoms become severe. Braak et al. have also reported that at post mortem occasional NFTs can be found in the brains of apparently healthy 30-year-olds [13].

Multiple studies have reported that the density of NFTs correlates more closely with cell dysfunction and symptoms than does Aβ plaque density. In-vivo Aβ PET imaging studies have demonstrated that Aβ deposition can occur 1–2 decades before the symptoms appear, levels approaching a plateau by the onset of cognitive symptoms. The Aβ cascade hypothesis posits that Aβ deposition is central to the pathology of disease, leading to a series of downstream events which cause tau hyperphosphorylation and aggregation, which results in neuronal dysfunction. Recent Aβ imaging studies support the Aβ cascade hypothesis to some extent [14], but it is now recognised that Aβ deposition alone cannot explain AD progression and pathogenesis. Thirty percent of elderly healthy subjects have significant levels of cortical amyloid deposition when imaged with ^11^C-PIB PET but manifest no overt symptoms [15]. Additionally, several high-profile anti-Aβ treatments have failed to halt or reverse the symptoms of AD [5].

Although the cascade hypothesis may be over simplistic, cognitively normal subjects who have brain Aβ deposition show cortical thinning [16] and have a higher risk of progressing to dementia [17, 18] so clearly amyloid aggregates are toxic to the brain. Aβ deposition, however, occurs at a slow rate over time, preceding neurodegenerative changes and cognitive deterioration [19], and levels correlate poorly with severity of cognitive symptoms. This suggests that strategies to remove amyloid could be most effective if used to protect early asymptomatic cases. In contrast, histopathological studies have shown that the presence of NFTs and neuronal loss increase in parallel with the duration and severity of symptoms [2]. While Aβ deposition plateaus early in the disease, NFT deposition and cell dysfunction continue to progress throughout the course of disease, correlating with symptom severity [3].

The emerging evidence, therefore, supports a complex pathological relationship between Aβ and tau aggregation that may also involve neuroinflammation in the form of microglial activation. This has been described as a ‘toxic pas de deux’ [20]. Histopathological comparisons of brains of non-demented, mildly demented and severely demented patients have shown that NFTs increase in all individuals with increasing age, but have a different distribution from that of Aβ plaques, indicating that the formation of tau and Aβ occurs independently. While NFT formation can occur early, the pathology progresses only slowly in isolation; however, if Aβ plaques are also present then their density increases rapidly. While tau and Aβ aggregation occur independently of one another, neither are sufficient alone to cause AD, and their pathologies may be interdependent [21].

This view is supported by animal studies. Injection of Aβ42 into the brains of transgenic mice expressing P301L pathological tau caused a 5-fold increase in NFT deposition compared with controls, indicating that the introduction of Aβ drove the tau pathology [22]. Furthermore, the offspring of transgenic mice expressing mutant tau crossed with mutant APP mice developed Aβ plaques at the same age, but had a significantly higher density of NFTs in the limbic system and cortex [23]. When brain extracts from APP transgenic mice were introduced into P301L tau transgenic mice, tau pathology was later identified not only at injection sites, but also in distant brain regions [24], indicating that introduction of Aβ triggered tau pathology and that tau aggregation was capable of ‘spreading’ across vulnerable neuronal networks in the brain. This may explain the predictable pattern of tau deposition throughout the cortex described by Braak staging. Aggregated tau is capable of leaving cells and causing normal tau to undergo aggregation and fibrillation [25]. For this reason, the transmission of tau aggregation has been likened to that of prions [26].

The presence of aggregated tau is a defining feature of several other neurodegenerative diseases, which include progressive supranuclear palsy [27], frontotemporal dementia related to chromosome 17 [28], argyrophilic grain disease (an age-related disease, caused by degeneration of argyrophilic grains, correlating with NFT deposition and cognitive impairment [29]), senile dementia of the NFT type [30], corticobasal degeneration and Pick’s disease [31]. Different ultrastructural forms of tau can cause different disease phenotypes. While normal human and Alzheimer brains contain equal amounts of 3R and 4R isoforms, Pick’s disease is characterised by aggregation of 3R isoforms into Pick bodies, while CBD, PSP, and argyrophilic grain disorders contain aggregated 4R isoforms as globose tangles in the case of PSP [1].

Chronic traumatic encephalopathy (CTE) is a progressive dementing neuropsychological illness in people who have suffered serial mild concussive brain injuries, which result in axonal injury. The condition has received significant attention in players of contact sports such as American football, rugby, and boxing, and also in horse riders who have frequent falls. It is characterised histologically by the deposition of tau in areas of axonal injury [32]. In one autopsy study of 85 people with mild and repetitive traumatic brain injury, compared with 18 controls, a clear and predictable range of NFT pathology across multiple regions was found, allowing for a grading system of tau pathology [33].

Table 1 shows the characteristic topographic features and distribution of tau aggregates in the common tauopathies. [31, 33–36]

**Table 1:** Common tauopathies with descriptions of the structure and distribution of tau aggregates

Tauopathy	Characteristics of aggregated tau	Location of aggregated tau	Other associations
Alzheimer’s disease (AD)	Intracellular NFTs containing both 3R and 4R inclusions; extracellular ‘ghost tangles’	Stereotypic progression from transentorhinal/entorhinal cortex, to amygdala, hippocampus, then widespread areas of cortex	Co-existence with extracellular β-amyloid plaques. Association with APOE4 allele
Frontotemporal dementia and Parkinsonism linked to chromosome 17	Tau cytoplasmic inclusions (3R, 4R and both 3R and 4R) in neuronal and glial cells	Widespread distribution	MAPT gene on chromosome 17q21-22
Progressive supranuclear palsy (PSP)	4R ‘globose’ inclusions, tufted astrocytes, oligodendroglial coiled bodies and threads	Subthalamic nucleus, basal ganglia, brainstem, and occasionally cortex	
Corticobasal degeneration (CBD)	4R ‘ballooned’ neurons and glial inclusions, astrocytic plaques, threads in grey and white matter, coiled bodies	Neocortical areas, and subcortical areas such as striatum	
Pick’s disease	Neuronal cytoplasmic Pick bodies (3R inclusions). Straight and twisted filaments	Dentate gyrus, followed by hippocampus, then cortex. Also seen in subcortical structures	
Argyrophilic grain disease (AGD)	4R spindle-shaped neuronal inclusions. Also pretangles, coiled bodies, astrocytic inclusions, and ballooned neurons	Amygdala, entorhinal/transentorhinal cortex, hippocampus	Often co-exists with other tauopathies such as AD. Association with H1 Haplotype of MAPT gene
Chronic traumatic encephalopathy	3R and 4R NFTs and prominent astrocytic tangles	Focal epicentres in the frontal lobe, then widespread cortical areas in a patchy distribution. High density in thalamus, mammillary bodies, brainstem, basal ganglia	Other pathologies can co-exist — β, TDP43, and α-synuclein
Primary age-related tauopathy (PART)	4R NFTs and ghost tangles	Restricted to mesial temporal lobe	Characterised by absence of amyloid plaque

## Tau PET imaging as a biomarker in AD

The National Institute of Ageing–Alzheimer’s Association (NIA–AA) Working Group Guidelines have emphasised the concept of AD as a spectrum or continuum of disease, consistent with the idea that pathophysiological changes of AD occur long before the onset of cognitive symptoms and ultimate dementia. Stages of disease can therefore be considered as an ‘AD Preclinical Stage’, reflecting the asymptomatic stage during which underlying pathology develops, and an ‘AD Clinical’ stage including mild cognitive impairment, when symptoms occur secondary to synaptic dysfunction and neuronal loss. The long prodrome has been identified as a key target time for disease-modifying therapy [37]. During the asymptomatic prodrome, imaging biomarkers can potentially be used to stage disease and follow its progression. Biomarkers in current use for detecting AD pathology are those reflecting Aβ deposition (reduced CSF Aβ levels and raised brain Aβ load) and markers of neurodegeneration (MRI atrophy, reduced glucose metabolism on FDG-PET, and raised CSF p-tau) [38]. These biomarkers have clinical utility in that they can predict risk of progression of mild cognitive impairment (MCI) to AD and health to MCI [39, 40] and give an indication of the sequence of pathological processes that occur in AD [39]. However, CSF measurement of tau requires a painful invasive spinal tap requiring a skilled operator, and does not provide critical information about the spatial distribution of tau. Aβ imaging provides useful information about the spatial and temporal distribution of Aβ deposition, but is not a marker of disease progression, due to the plateauing of plaque deposition.

There has been a global effort to identify a selective tau tracer to enable in-vivo PET imaging of NFT load as a method of identifying the spatial and temporal progression of tau pathology. As NFT density correlates with neuronal dysfunction and symptom onset, tau imaging should provide a valuable marker of disease progression. Simultaneous imaging of tau and Aβ aggregant load will promote a deeper understanding of the complex synergistic relationship between the two, helping to prove or refute much of the current speculation.

Tau PET imaging could be useful in clinical trials assessing the efficacy of anti-tau strategies. It will aid recruitment of subjects with a significant tau load (no longer relying on clinical assessment, which can be unhelpful, and markers of neuronal degeneration, which occur late in the course of disease), will provide proof of mechanism, and monitor tau clearance as an end-point. In the clinical setting, it can be used in the differential diagnosis of early dementias (differentiating between AD and non-AD pathologies), and also in differentiating between MCI and normal ageing [41–44].

## The search for a suitable tau tracer

While molecular imaging in dementia has been stimulated by the success of Aβ imaging, particularly using the Aβ tracer [^11^C]PIB, the identification of selective tau tracers has proved more difficult until the last few years. There are certain idiosyncrasies of the tau protein that need to be taken in consideration during tracer design. Tau is an inherently more complex and unpredictable protein, with multiple isoforms and many post-translational modifications. Therefore, tracers may bind specifically to a particular isoform or to multiple isoforms. Tau is an intracellular protein, so any ligand must cross the plasma cell membrane as well as the blood–brain barrier, which confers requirements about the molecular size and lipophilicity of the ligand. Furthermore, tau is present in the brain at much lower concentrations than Aβ, so a selective ligand will need to have a high binding affinity for tau over Aβ. This problem is confounded by the fact that Aβ and tau often co-exist in the same cortical areas, and both manifest β-sheet structure, which is where planar polyaromatic ligands tend to bind [41–45].

**Fig. 1:**
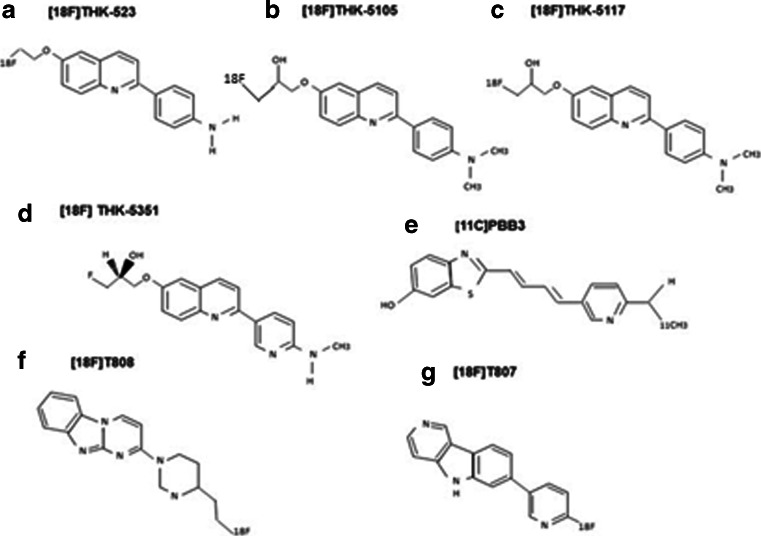
Chemical structures of current tau tracers. The chemical structures of: **a** [^18^F] THK-523, **b** [^18^F]THK-5105, **c** [^18^F] THK-5117, **d** [^18^F] THK-5351, **e** [^11^C] PBB3, **f** [^18^F]T808, and **g** [^18^F]-T807

Therefore, requirements of an ideal tau tracer are: (1) high sensitivity and selectivity for its target (20–50-fold selectivity is required for tau over Aβ), (2) low toxicity, (3) rapid uptake and clearance from the brain, and (4) no active brain metabolism. From a practical view, the radioactive half-life of the isotope used to label the ligand should also be taken into consideration. Use of ^18^F (half-life of 110 minutes) rather than ^11^C (half-life 20 minutes) can preclude the need for onsite production and allows longer imaging times, but increases the dosimetry. An ^11^C tracer may be preferred if multiple PET scans are to be performed, due to its lower dosimetry [41, 42, 44, 45]. Figure 1 shows the chemical structure of different tau tracers.

## [^18^F]FDDNP

2-(1-{6-[2-[^18^F]fluoroethyl)(methyl)amino]-2-naphthylethylidene)malononitrile (FDDNP) was developed as an amyloid marker by Barrio et al. in 2008 [46]. It is extracted well by the brain and shows moderate affinity for both amyloid plaques and neurofibrillary tangles, though the specific signal is low. FDDNP PET will separate groups of normal, MCI, and AD subjects by their levels of cortical uptake. The pattern of FDDNP uptake in AD reflects both amyloid and tau deposition, as signal is seen in both association cortex and hippocampus [47]. The uptake of FDDNP increases over time in AD and MCI due to increasing tau accumulation, so the tracer can be used to track disease progression. FDDNP PET has also been used to detect brain amyloid in Down syndrome [48] and dementia with Lewy bodies [49]. Drawbacks of FDDNP PET as a biomarker are its low sensitivity and selectivity. While it has been reported that [^18^F]FDDNP PET can predict progressive cognitive impairment in MCI [50], it is less sensitive than [^18^F]FDG PET for detecting disease progression. As a consequence, further searches for selective tau ligands were performed. A further consideration of FDDNP PET is the rapid metabolism and clearance of the tracer [51], and it does not reach a steady state for a long time. The optimal analysis method is therefore Logan graphical analysis using the cerebellum as a reference region, as used by Small et al. [52]. This requires long scanning periods (up to 125 minutes), making it a difficult tracer to use in clinical practice.

## THK compounds

The first tau-selective ligand, [^18^F]THK523, was identified by Okamura and colleagues at Tohuku University, after screening a series of quinolone and benzimidazole derivatives [59].

Pharmacokinetic studies showed excellent brain uptake and rapid clearance in mice, with no lipophilic metabolites and higher binding to tau over Aβ [59].[60] In-vitro studies of this ligand demonstrated binding to NFTs in AD brain sections and a higher affinity for tau fibrils than Aβ fibrils [61]. MicroPET studies in tau transgenic mice showed a correlation between in-vivo binding with subsequent histofluorescence [62]. However, while in-vivo testing in humans demonstrated selective tau binding, which correlated with the known tau distribution in AD, and a correlation of tracer uptake with impaired cognition, there was significant white matter retention which prevented accurate visual interpretation of signals, thus precluding its widespread use as a PET tracer [60]. Furthermore, it does not bind tau aggregates in non-AD tauopathies, further limiting its diagnostic utility [63].

Subsequently, the same researchers identified two further 2-arylquinoline derivatives, [^18^F]THK5105 and [^18^F]THK5117 which have superior binding affinity (K_i_ = 59.3nM for THK523, 7.8nM for THK5105, and 10.9nM for THK5117) and selectivity for tau in AD brains than [^18^F]THK523, as well as higher brain uptake and more rapid clearance. The tracer also has good penetration of the blood–brain barrier and no toxic effects [64]. In-vivo studies showed that there was higher cortical retention in AD patients compared with healthy controls, and retention correlated well with impaired performance on cognitive testing, and loss of brain volume [54].

**Fig. 2:**
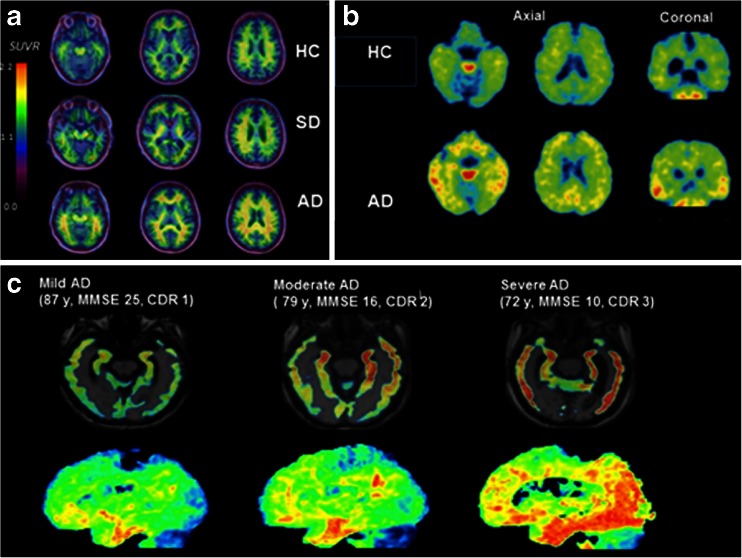
PET images using the [^18^F] THK family of tracers. **a** The first tau tracer, [^18^F] THK 523 in a healthy control, a subject with semantic dementia and an AD subject. There is increased tracer retention in the AD subject, but no difference between the control and SD Reproduced from Villemagne 2014 [53]. **b** [^18^F] THK5105 PET images in a 72-year-old healthy control (MMSE 29) and a 68-year-old AD subject (MMSE 20). Reproduced from Okamura 2014 [54]. **c** Tau tracer [^18^F]THK 5117 in a subject with mild, moderate, and severe AD, showing increasing retention of tracer as disease progresses from the medial, anterior, and inferior temporal cortex in mild AD, spreading to association areas in moderate AD, and throughout the neocortex in severe AD. Reproduced from Okamura 2014 [44]

[^18^F]THK5105 has a binding affinity to tau 25 times greater than that of amyloid, with peak brain entry higher than that for [^18^F}THK523, [^18^F]AV1451, [^18^F]T808, and [^11^C]PBB3 after 6 minutes. There is no obvious accumulation in the skull reported, but there is non-specific tracer retention in the brainstem, thalamus, subcortical white matter, probably due to binding to β-sheet structures in the myelin basic protein. This is not reported to be visually noticeable [54]. Compared to [^18^F]THK5117, [^18^F]THK5105 has a relatively slower clearance from the brain and higher lipophilicity, resulting in a lower signal to noise ratio [54]. Figure 2 shows [^18^F]THK523, [^18^F]THK5105, and [^18^F]THK5117 PET in different stages and types of dementia.

Brain uptake of [^18^F]THK5117 has been shown to have high affinity for tau in saturation binding assays, with nanomolar binding affinity [65]. It has been compared with that of [^11^C]PIB and [^18^F]FDG in subjects with MCI and AD. The authors noted a significant correlation between tracer retention of [^18^F]THK5117 and cognitive performance. In addition, they noted a different regional pattern of retention compared with [^11^C]PIB. The investigators reported lower [^18^F]THK5117 uptake in MCI compared with AD subjects, though both were raised compared to healthy controls, thus demonstrating the ability of the tau tracer to distinguish the spectrum of the Alzheimer disease process [66].

One report concerned three AD patients who had had [^18^F]FDG and [^11^C]PIB PET scans in life and donated their brains for subsequent post-mortem analysis, allowing in-vitro binding of [^18^F]THK5117 to be investigated by autoradiography. Binding of the tracer was highest in the mesial temporal region in all subjects, consistent with known tau pathology, but levels showed poor correlations with mesial temporal glucose metabolism and Aβ binding [67]. The authors concluded that tau imaging does not just mirror [^18^F]FDG PET findings.

Another 2-arylquinoline, [^18^F]THK5351 has also been recently developed which also shows high tau binding affinity in AD brains. [^18^F]THK5351 PET has been trialled in ten healthy controls and ten AD patients, while two other patients received [^18^F]THK5117 and [^18^F]THK5351 for a direct comparison. [^18^F]THK5351 had similar grey matter but lower white matter and brainstem retention than [^18^F]THK5117, potentially allowing for better tau visualization, while faster uptake and washout kinetics may facilitate kinetic modelling [68].

**Fig. 3:**
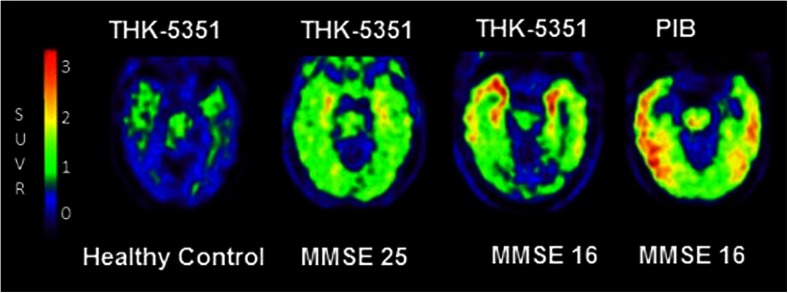
Novel tau tracer [^18^F]THK-5351 in different stages of cognitive impairment. PET images of [^18^F]THK-5351 in a healthy control, an MCI subject (MMSE 25), and an AD subject (MMSE 16). There is increasing tracer retention as disease progresses. In the AD patient, a [^11^C]PIB PET scan shows amyloid deposition in discrete separate areas of cortex. Courtesy of Nobayaki Okamura, unpublished work

The same group has also developed [^11^C]THK951, which has low lipophilicity (and therefore a higher signal to noise ratio), rapid brain uptake, and fast clearance. Uptake ratio in mouse brain was found to be superior to that of [^18^F]THK523, [^18^F]THK5105, and [^18^F]THK 5117, with slightly lower affinity to tau. In-vivo human testing has not yet been reported [69]. Figure 3 shows [^18^F]THK5351 in different stages of cognitive impairment.

## [^18^F] AV-1451 (T807) and [^18^F]T808

[^18^F]AV-1451 (T807) and [^18^F]T808 are tau-selective compounds recently synthesized by Hartmuth Kolb and his colleagues [70] and now tested in vivo [71]. [^18^F]T808 showed high binding affinity and good selectivity for tau over Aβ, with rapid uptake and washout in transgenic mice. MicroPET showed that it crossed the blood–brain barrier, with minimal white matter binding. However, studies in mice showed that ^18^F accumulated in bone, indicating defluorination was occurring, so the tracer has not been taken forward, even though it did not exhibit defluorination when tested in humans [72]. In-vivo tracer retention correlated closely with histological examination of tau deposition in another AD patient who died 2 weeks after PET images were obtained [73].

[^18^F]AV-1451 (T807) has demonstrated >25-fold selectivity for PHF-tau over Aβ (K_d_ = 14.6nM) on autoradiography and in vivo shows rapid brain extraction and washout, with no plasma metabolites entering the brain [74]. It is of low molecular weight (262.1 g/mol) and crosses the blood–brain barrier readily. The LogP of [18 F]AV-1451 is 1.67[74]. In mice, there was some accumulation in the bone noted, but the authors commented that radioactivity did not increase over time, and that brain homogenate in humans did not contain active metabolite [74]. Post-mortem validation of the tracer has shown high binding of the ligand to dystrophic neurites in AD [75]. In-vivo studies in humans show favourable [^18^F]AV-1451 uptake and washout kinetics, and tracer retention in AD mirrors the known distribution of tau in the brain. MCI cases showed lower uptake than AD patients, with tracer uptake patterns following Braak staging [55].

Pontecorvo et al. have reported preliminary findings in a PET study comparing Aβ and tau binding in subjects with MCI, AD, and cognitively normal controls. They noted highest tau deposition in patients with AD, followed by MCI, compared with low signal in normal controls. They also noted that cortical tau binding was significantly higher in Aβ-positive than in Aβ-negative individuals. In Aβ-negative individuals, hippocampal tau increased with age but no cortical deposition was detected [76].

Other studies have also shown increased binding of [^18^F]AV-1451 in MCI compared with controls which targeted all the association cortical areas. Worse cognitive performance (in terms of delayed recall) was associated with increased ligand retention in the entorhinal cortex [77]. Preliminary human studies using [^18^F]AV-1451 have been used to follow MCI and AD progression over relatively short periods of time (up to 19 months), and have shown significant rises in tau signals over time as symptoms progress, indicating the potential for tau tracers to detect disease progression [57].

[^18^F]AV-1451 (T807) also has the ability to distinguish variants of AD. In a patient with posterior cortical atrophy (PCA), binding was seen in primary visual and visual association cortices which correlated with decreased [^18^F]FDG uptake, whereas [^11^C]PIB uptake was globally elevated and showed no association with FDG metabolism [78]. The same group found that tau binding was increased in left parietal, temporal, and frontal regions in logopenic primary progressive aphasia [79].

[^18^F]AV-1451 PET has also detected increased signal in non-AD tauopathies such as PSP where the basal ganglia, thalamus, and frontal cortex were targeted. Its uptake has also been evaluated in patients with FTD, including the progressive aphasia and semantic dementia variants. The authors found that a symptomatic MAPT carrier showed increased ligand binding in the frontal, insular, and anterior temporal cortex, whereas in aphasic patients it was increased in left dorsolateral, prefrontal, and insular cortices. Patients with semantic dementia had highest uptake in the anterior temporal cortex with marked asymmetry [80].

Autoradiographic studies on brain specimens of patients with a range of disorders [AD, FTD-tau and PSP, CBD, Parkinsons disease (PD), dementia with Lewy bodies (DLB), and cerebral amyloid angiopathy (CAA)] have shown that while AD brains containing NFTs show high tracer binding, this is not evident in DLB, CAA, and FTD-TDP43. Fluorescent staining with disease specific tau antibodies revealed labelling of neurites and tangles in AD, PSP, CBD, and Pick’s disease, but not Lewy bodies or TDP43 [75]. Some preliminary work has shown binding of tracer in a distribution known to be compatible with PHF-tau distribution in PSP distinct from PD brains [81].

[^18^F]AV-1451 binding has been described in a retired American National Football League (NFL) player with cognitive decline and features suggestive of either CTE or PSP, and confirmed CTE based on binding patterns, demonstrating that tau imaging can help to differentiate between different types of dementia [82].

[^18^F]AV-1451 PET has also revealed insights concerning tau deposition in healthy cognitively normal individuals. Schultz et al. used Aβ and tau imaging to characterise in vivo the complex relationship between Aβ and tau pathologies. Seventy-five healthy elderly subjects were examined using longitudinal [^11^C]PIB and [^18^F]AV-1451 PET. A significant relationship was found between baseline Aβ burden and tau binding in the inferior temporal lobe, as well as an association between tau binding and the rate of Aβ accumulation, consistent with histology findings that Aβ pathology can influences tau ‘spreading’ throughout the cortex [83].

Sperling et al. compared Aβ with tau binding in cognitively healthy individuals, and showed that the presence of tau tangles on PET did not correlate with memory loss in healthy individuals unless Aβ plaques were also present. In the presence of plaques, however, there was a correlation between tau deposition and memory loss, again reinforcing the principle that the presence of Aβ accelerates tau pathology [84]. In cognitively normal elderly subjects, [^18^F]AV-1451 binding has been shown to correlate with levels of CSF tau [85].

**Fig. 4:**
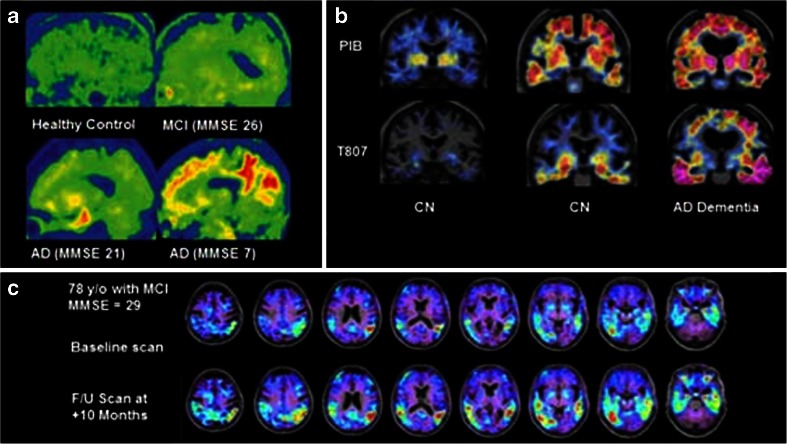
Novel tau tracer [^18^F]AV-1451 (previously [^18^F]-T807. **a** [^18^F]T807 in a healthy control (*top left*), through increasing severity of cognitive impairment to severe AD (*bottom right*). Increased tracer retention is seen as disease progresses, with widespread neocortical deposition in severe disease. Reproduced from Chien 2013 [55]. **b** PET images from two cognitively normal individuals, and one with AD dementia, with amyloid PET images on the *top row* ([^11^C]PIB) and tau PET images on the *bottom row* ([^18^F] T807). From left to right, increasing amyloid deposition is seen in the neocortex, as well as increasing tau in the inferior temporal cortices. Reproduced from Sperling 2014) [56]. **c** [^18^F] T807 in a subject with MCI at baseline, and after 10 months, showing a significant increase in tracer deposition in the temporal and parietal lobes. This indicates the clinical utility of tau imaging in detecting disease progression over relatively short time periods. Reproduced from Mark Mintun, 2015 [57]

Lockhart et al. used [^18^F]AV-1451 and[^11^C]PIB PET to study the effects of age on Aβ and tau aggregation in cognitively normal elders. They found significantly increased accumulation of tau in the basal ganglia, midbrain, hippocampus, and fornix of elderly normals, which extended to the neocortex as their age and the level of Aβ binding increased. The authors concluded that age and levels of Aβ binding could independently predict tau binding in healthy older people. Age predicted the level of tau accumulation in the medial temporal lobe, while levels of Aβ binding predicted tau deposition outside the medial temporal lobe [86]. Figure 4 shows [^18^F]AV-1451 (T807) in different stages of dementia and in longitudinal progression of disease.

## [^11^C]PBB3

Maruyama et al. have developed another family of ligands, the phenyl/pyridinyl-butadienyl-benzothiazoles/benzothiazoliums or PBBs, which bind strongly to NFTs in AD brains. In addition, ex-vivo examination of the brains and spinal cords of transgenic mice that had been injected with these compounds showed intense uptake in areas of tau accumulation [58]. [^11^C}PBB3 has a 40–50 times higher affinity for NFTs than for senile plaques, with affinity in the nanomolar range [58], readily crosses the blood–brain barrier (LogD = 3.3) [87], and is quickly washed out. There is minimal non-specific and white-matter binding [58].The tracer decays quickly to radioactive metabolites in preclinical models, but the radioactive metabolites have not been shown to enter the brain [87]. Nevertheless, simplified analysis methods such as Reference Tissue Models seem to agree with dual input compartment modeling [88].

**Fig. 5:**
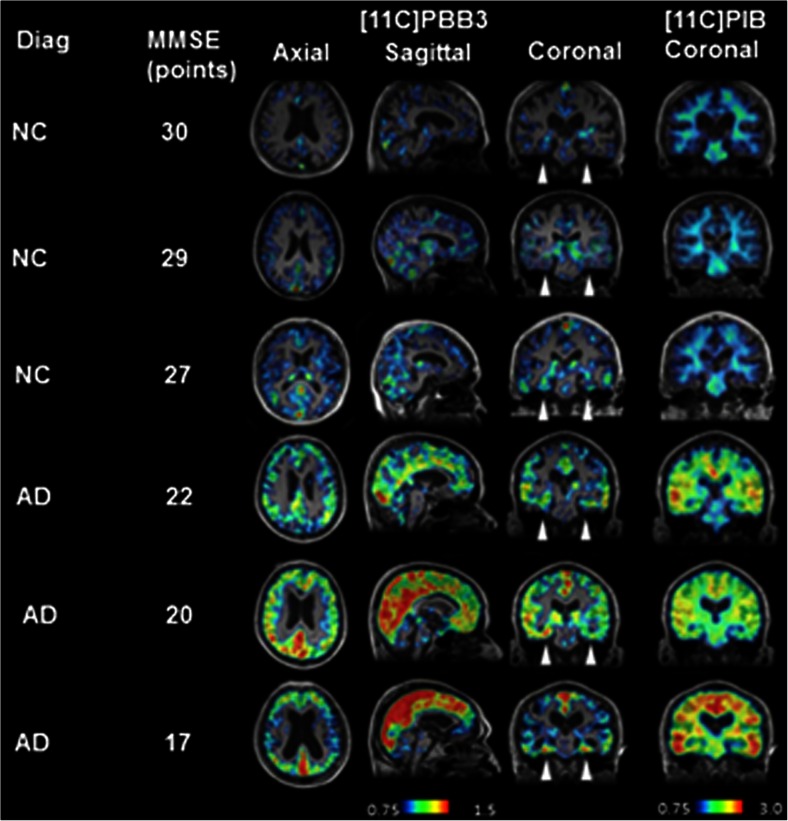
Tau tracer [^11^C]-PBB in differing stages of cognitive impairment. [^11^C]PBB3 and [^11^C]PIB in normal controls and AD patients with increasing severity of disease. The *arrowheads* indicate the hippocampi. While there is minimal tracer retention in the hippocampi of normal controls, there is increasing retention in the AD patients, especially as MMSE declines, with spread from the hippocampus to the neocortex, consistent with Braak staging. Reproduced from Maruyama 2013 [58]

[^11^C]PBB3 PET imaging of AD patients has shown increased tracer retention in the hippocampi in contrast to [^11^C]PIB. There was spreading of [^11^C]PBB3 binding throughout the cortex as the disease progressed [58, 89]. When [^11^C]PBB3 PET was performed in a patient with corticobasal degeneration, tracer retention was noted in the neocortical and subcortical structures, while [11C]PIB uptake was normal, highlighting the potential of tau tracers in non-AD tauopathies [58]. [^11^C] PBB3 has lower brain uptake than [^18^F]T807 or [^11^C]PIB due to this rapid metabolism and clearance, but the authors conclude that this may assist its selective binding to high-affinity, low-capacity sites on NFTs, compared to low-affinity, high-capacity sites on the more prevalent β-amyloid [87]. Figure 5 shows tau tracer [^11^C]-PBB in differing stages of cognitive impairment.

## Other compounds

Honer et al. have developed further compounds, RO6931643, RO6924963 and RO6958948, which are high-affinity binders at the [^3^H]T808 binding site on tau aggregates. All compounds have been noted to bind with high affinity and specificity to tau aggregates, and lack affinity to Aβ plaques. They also showed low non-specific binding in healthy brain tissues. In addition, there was macro- and micro-colocalisation of radioligand binding. Their pharmacokinetics showed rapid brain entry, washout, and safe metabolic patterns. These tracers are currently being tested in humans [90].

To date, several studies have used [^18^F]AV1451, which has demonstrated robust pharmacokinetics, high affinity for tau NFTs over amyloid, and minimal non-specific binding, as well as the ability to bind non AD tauopathies. Of the THK compounds, [^18^F] THK5117 has a very good pharmacokinetic profile, but with further data for [^18^F]THK5351 and [^18^F]THK951 awaited. [^11^C]PBB3 also offers good pharmacokinetics, with the ability to bind non-AD NFTs, but the short half-life of the ^11^C tracer may limit its use, and the rapid metabolism of the tracer may result in difficulty with data analysis.

## Conclusions

Recent years have seen exciting progress in the molecular imaging of dementia, and the tauopathies in particular. In-vivo tau imaging provides further information about the start and progression of the neuropathology of neurodegenerative disorders and, combined with amyloid imaging and FDG, it will be a promising biomarker, both clinically, in supporting differential diagnosis, and also in research, where it will help select appropriate patients and provide proof of mechanism and efficacy in clinical trials. While Aβ imaging plays a key role in the evaluation of dementia, the closer correlation of tau with cognitive impairment and neuronal dysfunction makes it more suitable as a biomarker of disease progression.

Several novel tau tracers are under development, and a number of phase 3 clinical studies are ongoing, with results keenly anticipated. In addition, further work involving tau, Aβ, and further pathologies, performed at different stages of the disease process will yield yet further insights into disease pathogenesis. These novel imaging targets give a real opportunity to diagnose dementia accurately, and to evaluate multi-targeted therapy much more efficiently.
